# Aldehyde Dehydrogenase, Liver Disease and Cancer

**DOI:** 10.7150/ijbs.42300

**Published:** 2020-01-22

**Authors:** Wenjun Wang, Chunguang Wang, Hongxin Xu, Yanhang Gao

**Affiliations:** 1 Department of Hepatology, The First Hospital of Jilin University, Jilin University, Changchun, Jilin, 130021, China.; 2 Department of Thoracic & Cardiovascular Surgery, Second Clinical College, Jilin University, Changchun, 130041, China.

**Keywords:** aldehyde dehydrogenase, gene polymorphism, liver disease, hepatocellular carcinoma

## Abstract

Acetaldehyde dehydrogenase 2 (ALDH2) is the key enzyme responsible for metabolism of the alcohol metabolite acetaldehyde in the liver. In addition to conversion of the acetaldehyde molecule, ALDH is also involved in other cellular functions. Recently, many studies have investigated the involvement of ALDH expression in viral hepatitis, alcoholic liver disease (ALD), non-alcoholic fatty liver disease (NAFLD), liver fibrosis, and liver cancer. Notably, ALDH2 expression has been linked with liver cancer risk, as well as pathogenesis and prognosis, and has emerged as a promising therapeutic target. Of note, approximately 8% of the world's population, and approximately 30-40% of the population in East Asia carry an inactive *ALDH2* gene. This review summarizes new progress in understanding tissue-specific acetaldehyde metabolism by ALDH2 as well as the association of *ALDH2* gene polymorphisms with liver disease and cancer. New research directions emerging in the field are also briefly discussed.

## Introduction

Our understanding of alcohol metabolism is well established at the molecular level. Alcohol metabolism mainly takes place in the liver and relies on two major nicotinamide adenine dinucleotide (NAD)-dependent enzymes, alcohol dehydrogenase (ADH) and aldehyde dehydrogenase 2 (ALDH2). Alcohol is first converted into acetaldehyde by ADH and cytochrome p450 2E1 (CYP2E1) via oxidative degradation, and the acetaldehyde is then oxidized to non-toxic acetate by ALDH and the coenzyme NAD or NADP for excretion 1. The further conversion of reactive aldehyde by ALDH family members offers cells with potential protection against radical oxidative species (ROS). However, the biological functions of ALDHs extend beyond detoxification, as the enzymes are involved in other biochemical processes, such as retinoic acid (RA) biosynthesis, catalysis of folate and amino acid and lipid peroxidation 2. Additionally, altered ALDH activity is associated with certain behavioral deficits 3, 4, endocrine disorders, cardiovascular and lung diseases 5, oral and gastrointestinal cancers, Fanconi anemia, and dermatitis 6.

In this review, we mainly summarized recent progress in understanding tissue-specific acetaldehyde metabolism by ALDH2 as well as the *ALDH2* gene polymorphisms associated with the pathogenesis of liver disease and cancer. Future research directions related to ALDH2 and liver diseases are also discussed.

## The Liver is the Major but Not the Sole Organ Responsible for Acetaldhyde Metabolism

Traditionally, it has been argued that the liver is the main organ responsible for ethanol metabolism 7. Overall, both expression and activity of ALDH2 in liver are much higher than those of other organs in wild-type mice 8. Surprisingly, a recent study found that after 3 hours of ethanol gavage, acetaldehyde levels in hepatocyte-specific *Aldh2* knockout mice are less than half of those in *Aldh2^-/-^* mice 8, suggesting other organs also contribute to acetaldehyde metabolism and clearance. Further studies in mice revealed that cumulative ALDH2 activity in multiple organs or tissues, such as white and brown adipose tissue, spleen, heart, and colon, may participate in acetaldehyde clearance 8.

## *ALDH* Genes and Polymorphisms

The human *ALDH* gene family consists of 19 putative members, and among the acetaldehyde dehydrogenase superfamily members, ALDH2 is enzyme that most efficiently catalyzes toxic acetaldehyde 9. *ALDH2* is located on chromosome 12 (12q24.12), is composed of 13 exons, and is approximately 44 kilobases in length. ALDH2 is abundantly expressed in both fat tissue and liver 8, in which there are two liver cytosolic and mitochondrial isoforms, and also expressed in kidney, lung, stomach and skin 10. Two ALDH2 isozymes are present in most Caucasians, but a single cytosolic isozyme without the mitochondrial isoform occurs in approximately 40-50% of the East Asian population. ALDH2 consists of four identical subunits (tetrameric), and each subunit contains triple functional domains of coenzyme or NAD^+^-binding (8-135, 159-270), catalysis (271-470) and oligomerization (140-158, 486-495) 11, 12.

Three isotype genes of* ALDH1* (*ALDH1A1, ALDH1A2,* and *ALDH1A3*) are located on 9q21.13, 15q21.3, and 15q26.3, respectively 13.* ALDH1* is expressed in both stem cells and differentiated cells. ALDH1 proteins, which are mainly found in cell cytosol in different tissues, oxidize retinal and aliphatic aldehydes. Cytosolic ALDH1A1 plays a role acetaldehyde oxidation and alcohol preference through mediating a γ-aminobutyric acid synthesis pathway 14. ALDH1B1, which is expressed at high levels in the liver and intestine epithelium, is an ALDH1 isoform that demonstrates high affinity to acetaldehyde only secondary to ALDH2 and catalyzes various aldehyde substrates of acetaldehyde and derivatives of lipid peroxidation 15, 16 (Table 1).

In addition, cytosolic ALDH (ascribed to ALDH1A1 expression) is highly expressed in hematopoietic stem and progenitor cells (HSPCs) and is relatively resistant to cyclophosphamide 17-19. High ALDH activity in hematopoietic progenitor cells offers a biomarker for easy identification and isolation of viable hematopoietic progenitor cells with the new Aldefluor substrate assay 17. This high ALDH activity marker can also be used for isolating liver progenitor cells (LPCs) from murine and human liver 20. It is believed that LPCs with high ALDH1 expression provide a cell source for regenerating hepatocytes for repair of injured liver in vivo and for toxicology studies* in vitro* 20. However, a recent study demonstrated that deletion of the *Aldh1a* gene does not affect LPC proliferation or hepatocellular carcinoma (HCC) progression 21.

### Effects of Mutant ALDH2 Protein on Acetaldehyde Metabolism

Single nucleotide polymorphisms (SNPs) have been identified among genes encoding ADH, microsomal enzyme CYP2E1 and other alcohol-metabolizing enzymes including ALDH2. A total of 84 SNP sites in the human *ALDH2* gene have been reported 22.

Furthermore, rs671 is the most studied SNP in the *ALDH2* gene, and mitochondrial ALDH2*2 is a variant with nucleotide mutation that results in replacement of glutamate with lysine (K) at residue 487 (E487K) in the oligomerization domain. This change alters the enzyme activity and metabolic efficiency *in vivo* 11. In addition, when the leader sequence in the mitochondrial isoform is included, position 487 of the mature protein is actually counted as amino acid position 504; thus, this mutant is also known as ALDH2*504 Lys 23. The E487 change impacts the dimer and tetramer formation, as E487K disrupts the conformational structure in the polymorph, resulting in loss of the ability for di- or tetramerization 12. The *ALDH2 E487K* variant shows decreased flexibility at the domain of catalysis and coenzyme binding and increased flexibility at the domain of oligomerization 12. Upon replacement of wild-type E487 with lysine, the variant K487 interferes with the local secondary structure when dimerized, conferring an unstable dimer interface. The K residue at the amino acid 487 position cannot form a hydrogen bond with arginine 264 at the same subunit or arginine 475 in the dimer partner, damaging the NAD^+^-binding site, particularly in the αG helix of the dimeric interface 24. The E487K mutant has altered kinetic properties; i.e., the K_m_ value for NAD^+^ at a physiological pH is increased by >150-fold, the K_ia_ (representing the dissociation constant for NAD^+^ from the enzyme) is increased by nearly 50-fold, and the K_cat_ is decreased by 2-10-fold compared to the recombinant native enzymes 25. Furthermore, the rate-limiting step is shifted from hydride transfer or coenzyme dissociation to the NAD^+^-binding enzyme; coenzyme binding becomes slower and weaker 24, 25.

In terms of the ingestion of alcohol, individuals who have the homozygous ALDH2*2/*2 genotype lose ALDH2 activity and cannot metabolize acetaldehyde, while those with the heterozygous ALDH2*1/*2 genotype have a reduced ability (loss of >90% of activity) to metabolize acetaldehyde. Consequently, acetaldehyde excessively accumulates in cells, resulting in a flushing response, Asian glow or flush accompanied by headache, perspiration, tachycardia, palpitations, nausea, and sleepiness after consumption of alcohol. A more severe reaction is seen in those individuals with the homozygous null gene, compared with that in heterozygous individuals with the incomplete knockout 26.

### Distribution of ALDH Alleles in Different Populations

Human ALDH2*2 is the most common variant among *ALDH* gene members and occurs in an estimated 560 million people or an average 8% of the global population. However, the ALDH2*2 positive percentage varies from race to race, being as high as 40% in Asians and <5% in European and African populations 27, 28. Genetic studies have suggested that the ALDH2*2 variant can be traced back to a Han Chinese ancestor in central China and that it continued spreading in many regions of East Asia over several millennia. Populations with a high percentage of individuals positive for the ALDH2*487 Lys allele are largely localized in the South Fujian province and East Guangdong province in southeastern China, and in Chiba (34.1%), a central region in Japan 23. Continuous migration and population growth of Han Chinese expanded the ALDH2*487 Lys genotype to neighboring regions 23. A study investigating 4018 Chinese Han adults found that the frequencies of ALDH2*1/*1 (GG), ALDH2*1/*2 (GA), and ALDH2*2/*2 (AA) were 68.67%, 28.67%, and 2.66%, respectively 29.

### Screening Strategy for ALDH2

High-resolution melting analysis is considered a sensitive closed-tube approach to determining SNPs of* ADH1B* and *ALDH2.* This assay can be completed in 2 hours, costs only $0.50, and is suitable for population screening 30. In addition, polymerase chain reaction-based restriction fragment length polymorphism analysis can also distinguish two polymorphic alleles for both the* ADH1B* and* ALDH2* genes within 1.2 hours, and thus, can be used for both small- and large-scale analyses 31.

Non-assay-based investigations, i.e., a simple questionnaire of presence or absence of current and past flushing, can be used for identifying inactive *ALDH2* variants in population-based epidemiological studies 32. A note of caution is that, despite the presence of ALDH2*1/*2 or ALDH2*2/*2, individuals with a semi-active or knockout form of *ADH2* (e.g., ADH2*1/*1 or ADH2*2/*1) may not experience obvious flushing after light drinking or facial flushing may be diminished in individuals with a long or heavy drinking history 32. Another study found that the sensitivity and specificity of the modified alcohol flushing questionnaires were 95.1% and 76.5%, respectively, in healthy male subjects, and 78.9% and 82.1%, respectively, in subjects who participated in a gastric cancer screening program or received esophagogastroduodenoscopy 33.

## ALDH and Liver Diseases

### ALDH and Viral Liver Diseases

Chronic alcohol-related liver injury and viral hepatitis are two major categories of chronic liver disease, and often both etiologies occur in the same liver, accelerating liver disease progression 34. The prevalence rates of hepatitis C virus (HCV) infection in the US, European countries and Japan were reported to be 1.13%, 1.10%, and 1.56%, respectively, and these rates are higher than that of 0.72% in China 35. A national household survey conducted between 1999-2002 in the United States showed that adults who were HCV RNA-positive had heavier alcohol intake, were almost three times more likely to consume >1 drink per day (35.3% vs. 13.5%, *P*=0.003), and were almost 8 times more likely to consume >3 drinks per day (19.2% vs. 2.4%, *P*=0.010) compared with other adults 36. Alcoholic individuals who are also intravenous drug users are at a much higher risk for contracting HCV infection. According to the data from the 2003-2010 National Health and Nutrition Examination Survey, the median number of US residents with HCV infection was estimated to be 4.6 million (range, 3.4-6.0 million), and of these, at least 3.5 million (range, 2.5-4.7 million) were viremic 37. The combination of HCV infection and alcohol abuse accelerates chronic liver disease to end stage 38. On the other hand, the prevalence of chronic hepatitis B virus (HBV) infection in China is high, with an estimated 6.52% of the general population being hepatitis B serum antigen (HBsAg) positive 35. Our recent study showed that a history of excessive drinking was identified in 26.5% of chronic hepatitis B patients without cirrhosis, in 35.6% of those with HBV-associated cirrhosis, and in 41.8% of those with HBV-associated HCC 39. Lin *et al.* found a good geographical correlation between populations who lived in HBV endemic regions and carried the ALDH2*2 alleles 40. Globally, the highest liver cancer incidence and HBV prevalence are detected in Eastern Asia and Africa 40. Research has also found frequencies of ALDH2*1/*2 or ALDH2*2/*2 of 29%, 35.3%, 28.0%, and 37.9% in healthy controls, chronic hepatitis B patients without cirrhosis, patients with HBV-associated cirrhosis, and patients with HBV-associated HCC, respectively 39.

**Figure 1:**
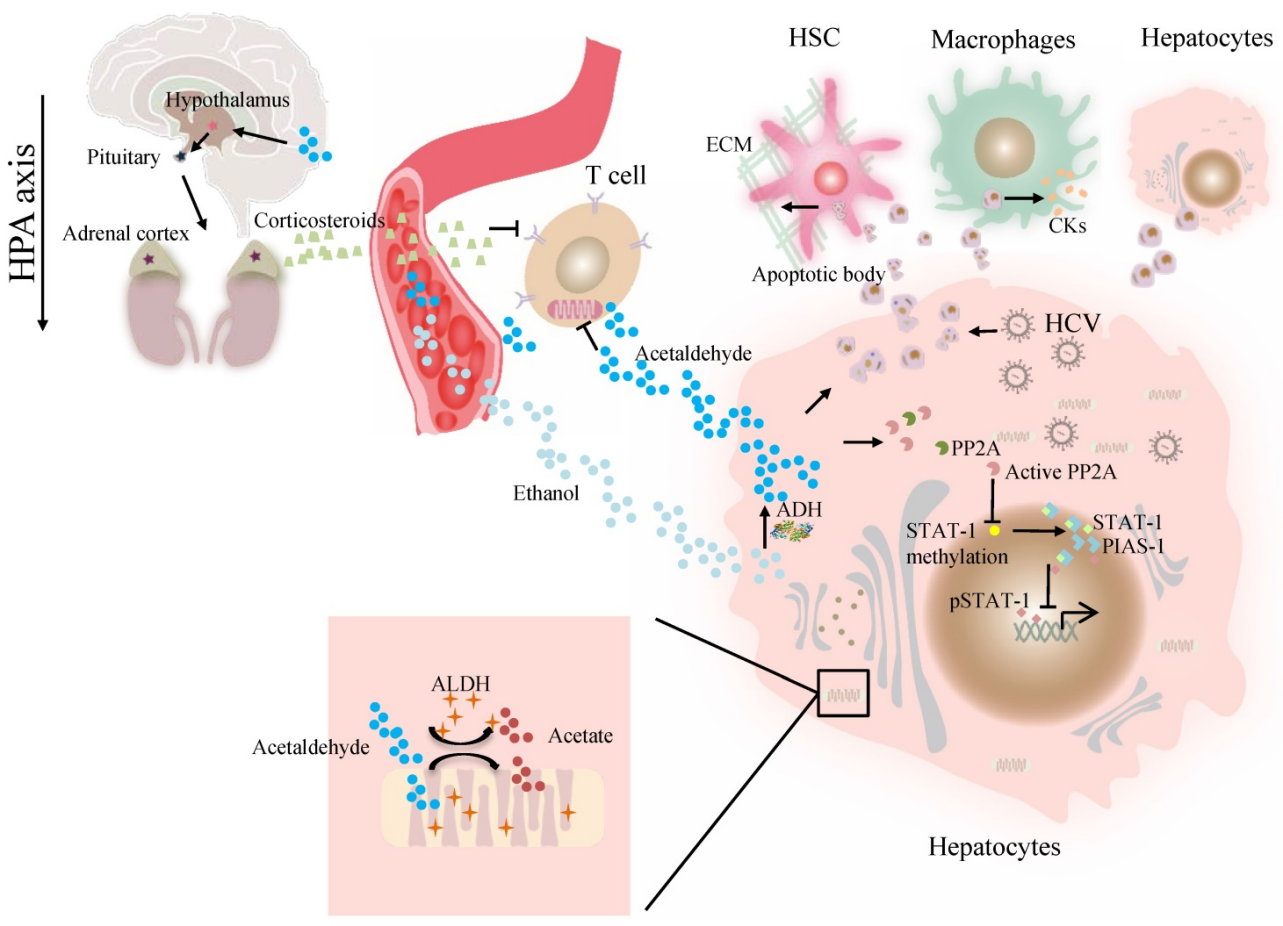
The effect of ALDH in viral liver diseases. Ethanol is converted to acetaldehyde by the cytosolic enzyme ADH and the microsomal enzyme CYP2E1. Then acetaldehyde is converted to acetate by ALDH. Acetaldehyde directly suppresses cytokine production in T cells through the inhibition of aerobic glycolysis or stimulation of corticosterone release through HPA axis. Acetaldehyde also activates PP2A, leading to reduced STAT-1 methylation and formation of the PIAS-1-STAT-1 complex. Consequently, pSTAT-1 attachment to DNA is decreased, and ISG activation is inhibited. Acetaldehyde induces apoptosis in HCV -infected hepatocytes. Destruction of HCV-infected cells via AB release infectious HCV virions for *de novo* infection Kupffer cells, HSCs, and hepatocytes engulfed AB, potentially inducing of hepatic inflammation, fibrosis and apoptosis. Abbreviations: ALDH, aldehyde dehydrogenase; ADH, alcohol dehydrogenase; AB, apoptotic body; CYP2E1, cytochrome P-450 2E1; HPA, hypothalamic-pituitary-adrenal; ; HCV, hepatitis C virus; HSCs, hepatic stellate cells; ISG, interferon-stimulated genes; PP2A, protein phosphatase 2A; PIAS-1, protein inhibitor of activated STAT-1; STAT, signal transducer and activator of transcription.

At present, the association of ALDH genotype and viral hepatitis at the molecular level remains largely unexplored. The major difficulty is a lack of valid models in which a liver can support viral infection and alcohol metabolism that resembles liver injury caused by alcohol consumption and viral infection in alcoholic patients with chronic hepatitis C 41. Our previous work demonstrated that alcohol-fed *Aldh2*^-/-^ mice were less sensitive to concanavalin A-induced T-cell hepatitis than wild-type mice 42. Further studies suggest that acetaldehyde directly suppresses cytokine production in T cells through the inhibition of aerobic glycolysis or stimulation of corticosterone release, which contributes to the occurrence of suppressed T-cell hepatitis in ethanol-fed *Aldh2*^ -/-^ mice 42. Cho *et al.* suggested that the ADH/ALDH pathway also exerts a potent antiviral activity, likely mediated through the catalysis of retinol (ROL) and RA biogenesis, leading to the expression of interferon-stimulated genes (ISGs) 38. Furthermore, acetaldehyde activates protein phosphatase 2A (PP2A), leading to reduced signal transducer and activator of transcription (STAT)-1 methylation and formation of the protein inhibitor of activated STAT-1 (PIAS-1)-STAT-1 complex 43. Consequently, pSTAT-1 attachment to DNA is decreased, and ISG activation is inhibited, compromising antiviral function in HCV-infected hepatocytes 43. The ISG pathway consists of more than 300 antiviral molecules that synergistically exert innate immunity. Betaine, a compound that functions as a methyl donor, can mitigate the acetaldehyde-mediated inhibition effect on IFN signaling, and thus, can be used to relieve the negative impact of accumulated acetaldehyde in HCV+ alcoholic patients 43. The antiviral activity of the ADH/ALDH pathway may be compromised even by a physiological alcohol concentration in alcoholic individuals, likely through alcohol-ROL metabolic competition 38. A mechanism for acetaldehyde exposure-induced liver injury in HCV-infected cells was proposed by Ganesan et al., who found that acetaldehyde is continuously generated in the acetaldehyde-generating system (AGS), resulting in a transient increase in HCV RNA, which subsequently recedes to normal or lower levels when a significant number of HCV-infected cells undergo apoptosis 41. Acetaldehyde is also associated with higher miR-122 and miR-34a expression in HCV-infected hepatocytes, leading to higher HCV replication, and consequently, apoptosis due to robust HCV replication and accumulation of viral products. Destruction of HCV-infected cells via apoptosis bodies results in the release of infectious HCV virions for* de novo* infection that expands the HCV infection in the liver, aggravating liver injury and delaying HCV clearance 41. The apoptotic bodies engulfed by Kupffer and hepatic stellate cell (HSC) potentially induce hepatic inflammation and fibrosis 41 (Figure 1). In summary, future studies that focus on identifying novel mechanisms and devising therapeutic strategies for these viral liver diseases in ALDH2-inactive individuals are warranted.

### ALDH and Alcoholic Liver Disease (ALD)

As many developing countries have experienced significant economic expansion and improved living standards, a byproduct has been an alarming rise in alcohol consumption and subsequently ALD 35. The prevalence rates of ALD were reported to be 4.5%, 6.2% 6%, and 1.56-2.34% in China, the US, Europe, and Japan, respectively 35, 44-46.

Alcohol flushing response can be a defensive mechanism, and it may deter alcohol consumption and reduce ALD in individuals with ALDH2*487 Lys 47, 48. A meta-analysis of 12 studies found that individuals with ALDH2*1 allele are connected with a higher frequency of alcoholic liver cirrhosis (ALC) compared with those with ALDH2*1/*2 or ALDH2*2/*2 genotype 49. In addition, a single-center study from Beijing 302 Hospital in China reported that only 2.3% of ALD patients had the *487 Lys* allele compared with 14.5% of healthy controls (281 cases and 535 controls; odds ratio [OR] = 0.13, 95% confidence interval [CI]: 0.07-0.24) 47. Similarly, Tanaka *et al.* observed the ALDH2*1/*1 genotype more frequently in an alcoholic population and ALD patients in Japan, than in control subjects (95.6% and 80.6% vs. 39.4%, *P*<0.01) 50. Chao* et al.* reported that patients with alcoholic cirrhosis and alcoholic dependence had a significant lower frequency of the ALDH2*2 allele than did healthy controls (9%, 6% vs. 30%, *P*<0.005; n= 27, 50, and 50, respectively) 51. They also reported a significant higher frequency of the ALDH2*1 allele in patients with alcoholic cirrhosis vs healthy controls (93% vs. 71%, *P*<0.001; 75 cases and 100 controls) 52. Lee *et al*. reported a significantly higher frequency of the ALDH2*1 allele in patients with alcoholic cirrhosis (96%, n=56) and in alcoholic individuals without evidence of liver disease (98%, n=52), compared with nondrinkers (74%, n=64, *P*=0.001) in Korea 53. However, the observed protection against ALD by the ALDH2*2 allele can wane over time. Edenberg* et al.* found that the percentage of alcoholic Japanese individuals with the ALDH2*2 allele increased from 2.5% to 13% during the period of 1979 to 1992, indicating that this allele's protective function can be overcome by more alcohol consumption, which increases alcohol tolerability 54. Recent research demonstrated that hepatocyte-specific *Aldh2* knockout mice reduced excessive alcohol preference rapidly but not light to moderate alcohol concentrations; therefore, targeting liver ALDH2 represents a promising approach to prevent heavy drinking with limited systemic side effects 8. Culturally, people are encouraged or challenged to drink more alcohol in social events, and sometimes individuals with face flushing may not be able to escape or decline this drinking binge because any reluctance in drinking is considered rude 55.

Interestingly, Kwon* et al.* reported that *Aldh2*^-/-^ mice become more susceptible to alcohol-induced liver inflammation but more resistant to alcohol-induced steatosis. One possible reason for these diverse effects is that malondialdehyde-acetaldehyde adduct (MAA)-mediated paracrine activity stimulates interleukin (IL)-6 expression in Kupffer cells, which activates STAT3 in hepatocytes and then attenuates the transcription of SREBP1c (sterol regulatory element-binding protein 1c) 56. Hepatocellular SREBP1c mRNA levels and the expression level of acetyl-CoA carboxylase-1, a target gene of SREBP1c and fatty acid synthase were significantly lower in the alcohol-fed *Aldh2*^-/-^ mice than in wild-type mice 56. Chaudhry *et al.* reported that *Aldh2*^+/-^ mice were prone to alcohol-induced gut barrier dysfunction and fatty liver, because acetaldehyde damages the epithelial barrier and increases the permeability of the epithelial layer in the intestinal tract 57. One study suggested that overexpression of ALDH2 is protective through the attenuation of chronic alcohol-induced liver damage and apoptosis (caspase 3 activity) 58. Zhong *et al.* considered mitochondrial ALDH2 a promising therapeutic target for ALD, based on their finding that a selective small-molecule ALDH2 activator, N-(1,3-benzodioxol-5-ylmethyl)-2,6-dichlorobenzamide (Alda-1), can accelerate aldehyde clearance and reverse hepatic steatosis and apoptosis in mice through pharmacological activation of ALDH2 59. In addition, alcohol intake induces mitochondrial damage, one of the hallmarks of ALD 60. A mitochondria-targeted lipophilic ubiquinone (MitoQ) was reported to expedite clearing of acetaldehyde and lipid aldehyde and to restore ALDH activity by blocking post-translational oxidative/nitrosative modification of mitochondrial ALDH2 in mice 60. However, activation of ALDH2 by Alda1 or MitoQ may increase drinking and exacerbate ALD; thus the treatment of ALD patients with ALDH2 activators may not achieve significant beneficial effects 61. Overall, alcohol consumption is one of the major avoidable risk factors for ALD, and abstinence is considered foundational for successful ALD treatment.

### ALDH2 and Non-Alcoholic Fatty Liver Disease (NAFLD)

An often underappreciated but important trend is that the incidence of metabolic liver diseases continues increasing worldwide 62-64. A global meta-analysis of studies found that the global NAFLD prevalence in 8,515,431 individuals diagnosed by imaging findings was 25.24% (95% CI: 22.10-28.65), and the prevalence was highest in the Middle East and South America and lowest in Africa 65. The estimated regional NAFLD percentages in Asia and Israel were 52.34 per 1,000 (95% CI: 28.31-96.77) and 28.01 per 1,000 person-years (95% CI: 19.34-40.57), respectively 65. The prevalence rates of NAFLD were found to be 22.4%, 24.13%, 23.71%, and 25% in China, the US, Europe, and Japan, respectively 35, 63, 64, 66. NAFLD covers a spectrum of liver pathologies from fatty liver to non-alcoholic steatohepatitis (NASH), fibrosis to cirrhosis, liver failure and HCC. Day *et al.* proposed a 'two hit' model for NAFLD pathogenesis 67. The 'first hit' produces steatosis, and then the 'second hit' includes oxidative stress to initiate significant lipid peroxidation to injure liver cells 67. Endoplasmic reticulum stress occurs in the course of NAFLD and contributes to the production of ROS, which can provoke oxidation of polyunsaturated fatty acids to form lipid peroxidation products, such as 4-hydroxy-nonenal (4-HNE) 68.

Of particular interest, NAFLD at the progressive stages may also impact alcohol metabolizing enzymes and alcohol metabolism processes. Li *et al.* reported that ALDH4A1 mRNA was significantly decreased in two NASH groups (fatty and non-fatty: >5% and <5% of hepatocytes with fat deposition), in contrast to no significant changes in the mRNA levels of other alcohol-related enzymes 69. However, the protein levels of ALDH2 were significantly increased in both NASH groups, whereas the ALDH1A1 and ALDH1B1 protein levels were significantly reduced. Furthermore, significant oxidative stress and reduced ALDH activity were suggested by the significant accumulation of 4-HNE protein adduct in NASH 69. 4-HNE is a covalent modification of an ALDH2 active site peptide and is reported to be a potent irreversible inhibitor of ALDH2, suggesting 4-HNE adduct formation may inactivate ALDH2 70. As reported, Alda-1 may exert a range of biologic functions from enhancing the detoxification activity of ALDH2 and attenuating hepatic fatty content including triglycerides to reducing the formation of 4-HNE protein adduct in apolipoprotein E-knockout mice (apoE^-/-^) 71. However, a systematic transcriptome analysis revealed that the expression of alcohol-metabolizing enzymes, including ADH, ALDH (ALDH1A1, ALDH1B1, ALDH3B1, ALDH4A1, ALDH7A1, and ALDH9A1), CYP2E1, and CAT, was higher in NAFLD livers (72 NAFLD patients and 7 controls) 72. Overall, these findings support a role for alcohol-metabolizing enzymes in NAFLD pathology, and further studies are needed to clarify the effects of NAFLD on alcohol metabolism and how alcohol affects NAFLD pathogenesis.

Although alcohol consumption and NAFLD commonly exist, the data on the effects of moderate alcohol drinking on NAFLD progression remain controversial 73-76. Data from the National Health and Nutrition Examination Survey conducted from 1988 to 2010 revealed that modest alcohol consumption (0.5-1.5 drinks or 7-21 g/day) is associated with decreased mortality among patients with NAFLD 73. The modest drinking has a protective effect partly via decreases in cardiovascular disease mortality and metabolic syndrome 73, 76. In contrast, a large-scale cohort study of 58,927 Korean patients with NAFLD demonstrated that even moderate drinkers (10-29.9 g/day) exhibited an increased tendency to progress to fibrosis compared with nondrinkers 74. Further investigation should be conducted to figure out whether moderate alcohol consumption can become a lifestyle intervention in the treatment. In addition, the effects of ALDH2 polymorphism on NAFLD with or without alcohol drinking have not been carefully studied.

### ALDH and Liver Fibrosis

An estimated 7 million (or 0.51%) Chinese individuals suffer from liver cirrhosis, which is accompanied by 460,000 new cases of liver cancer each year. The reported prevalence rates of cirrhosis were 0.27%, 0.10%, and 0.31-0.39% in the US, Europe, and Japan, respectively 35. In more than 80% of cases, HCC is preceded by fibrosis or cirrhosis, implicating liver fibrosis as a premalignant lesion 77. Previous studies reported that inactive ALDH2 and super-active ADH2 alleles were associated with liver fibrosis as a result of increased acetaldehyde accumulation in hepatocytes 78. In addition, a recent study showed that the fibrotic liver has significantly higher activity of total ADH, ADH1 and ADH2 compared with the normal liver (*P*<0.001), while ALDH activity was higher, but insignificantly (median, range = 0.12, 0.02-0.32 nmol·min^-1^·mg^-1^ protein vs. 0.15, 0.03-0.35 nmol·min^-1^·mg^-1^ protein, *P*<0.01) 79.

*Aldh2*^-/-^ mice are also more susceptible to ethanol plus carbon tetrachloride -induced liver injury, inflammation and fibrosis through acetaldehyde and its derived adducts 56. Increasing evidence indicates that acetaldehyde enhances HSC activation in fibrogenesis* in vitro* 56, 80. In response to liver injury, HSCs may be activated and converted to a myofibroblast-like cell morphology by losing the quiescent fat-storing phenotype, increasing proliferation and fibrillar collagen production, and showing pro-inflammatory activity 77, 81. Activated HSCs represent the main source of excessive extracellular matrix (ECM) production and are likely involved in alcohol-induced fibrosis as well 80. Acetaldehyde and ROS are originally generated in hepatocytes but may be taken up by HSCs in a paracrine fashion, where acetaldehyde promotes collagen I synthesis 78, 81. Promoters for both collagen α_1_ (I) and collagen α_2_ (I) (COL1A2) contain acetaldehyde-responsive elements in the different transcription factor binding sites 82. Reyes-Gordillo* et al.* proposed that the acetaldehyde-induced up-regulation of COL1A2 transcription is mediated by two distinct mechanisms. The first mechanism is transforming growth factor beta 1 (TGF-β1)-independent, and it mainly eliminates repressors of COL1A2 (such as Ski and SMAD7) and phosphorylates SMAD3 (up to about 6 hours). This step occurs swiftly and also transiently 78, 83. The second mechanism features a sustained acetaldehyde-mediated upregulation of TGF-β1 expression. Both phosphorylation and nuclear translocation of SMAD3/4-containing complexes are then induced to lead to binding of the COL1A2 promoter by acetaldehyde and TGF-β, which induces the production of collagens for fibrogenesis 83. Moreover, acetaldehyde may upregulate the interstitial collagenase matrix metalloproteinase (MMP)-2 gene and downregulate the fibrillary collagenase MMP-1, resulting in a sclerotic matrix with substitution of the normal ECM components 81.

**Figure 2:**
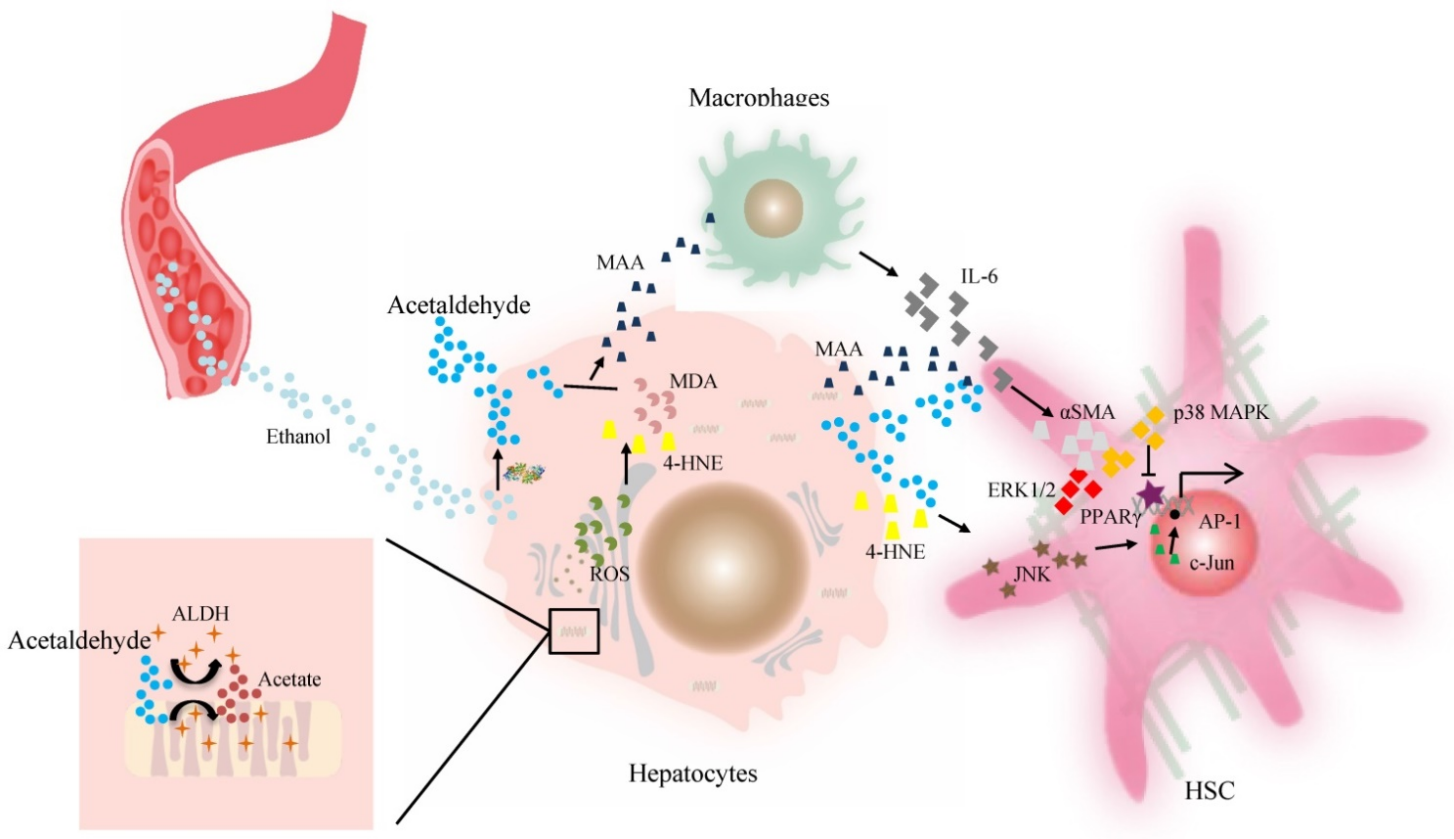
The effect of ALDH in liver fibrosis. Acetaldehyde can be taken up by HSCs in a paracrine fashion. Both acetaldehyde and 4-HNE can promote collagen I synthesis in HSCs through JNK/ERK/ AP-1 signaling. MAA adduct stimulate Kupffer cells to produce a higher level of IL-6, which in synergy with acetaldehyde or MAA, can enhance the expression of αSMA protein and promote HSC activation and proliferation via p38 MAPK and ERK1/2 activation. The PPARγ transcriptional activity is inhibited by acetaldehyde through rapid activation of PKCδ and the ERK1/2 pathway, contributing to activation of HSCs. Then activated HSCs are the main source of excessive ECM production. Abbreviations: ALDH, aldehyde dehydrogenase; AP-1, activating protein-1; ECM, extracellular matrix; HSCs, hepatic stellate cells; 4-HNE, 4-hydroxy-nonenal; IL, interleukin; JNK, c-Jun amino-terminal kinase; MAA, malondialdehyde-acetaldehyde; PPARγ, peroxisome proliferator-activated receptor gamma; αSMA, α-smooth muscle actin.

Acetaldehyde-derived adducts may also promote liver fibrosis. The aldehyde molecule tends to be unstable and quickly reactive with cellular components and generates adducts without proper function. This step appears to be key in alcohol-induced fibrosis. The progression of liver fibrosis correlates well with elevated levels of acetaldehyde-protein and acetaldehyde-lipid adducts of malondialdehyde (MDA), 4-HNE, and mixed MAA adducts in both alcoholic patients and animal models 78, 84. A suggested mechanism for aldehyde-mediated activation of HSCs includes that 4-HNE activates p46 and p54 isoforms of c-Jun amino-terminal kinase and activating protein-1 in HSCs 84. However, as reported, HSCs isolated from the liver of a rat model of cirrhosis can metabolize 4-HNE at a higher efficiency, which contributes to their higher ALDH activity in* vitro* 80, compared with HSCs from the normal rat liver. Finally, Kupffer cells stimulated by higher levels of MAA adduct produce a higher level of IL-6, which in synergy with acetaldehyde or MAA, can enhance the expression of α-smooth muscle actin protein and promote HSC activation and proliferation via p38 MAPK and ERK1/2 activation 56, 85, 86. Then peroxisome proliferator-activated receptor gamma (PPARγ) transcriptional activity is inhibited by acetaldehyde through rapid activation of PKCδ and the ERK1/2 pathway, whereas the depression of PPARγ transcriptional activity is associated with activation and proliferation of HSCs 81, 87. ALDHs catalyze retinaldehyde to RA, a strong morphogen that triggers cell differentiation and proliferation during development 20, 88. RA is also implicated in fibroblast proliferation and expression of ECM collagen in activated HSCs, which contributes to liver fibrosis 88, 89 (Figure 2). Therefore, individuals with the dominant inactive ALDH2*2 gene should be informed of their higher risk for liver inflammation and fibrosis after moderate or heavy drinking.

### ALDH and HCC

Liver cancer ranked as the fifth most common cancer and the second most malignant cancer for cancer-related mortality in the world in 2015 90. The reported prevalence rates of liver cancer were 0.03%, 0.01%, <0.01%, and <0.01% among the general population in the China, US, Europe, and Japan, respectively 35. Chronic HBV infection is the leading etiology for HCC not only in HBV endemic regions, but also globally. The second most common etiology is ALD using the measure of disability-adjusted life-years 90. Because carcinogenesis implicates environmental and genetic factors, it is important to gain a perspective on the correlation of genetic variations in alcohol-metabolizing enzymes with liver cancer occurrence.

ALDH is associated with HCC risk, progression, and prognosis. A dose-dependent association between the cumulative amount of alcohol consumption over time and HCC risk in individuals with the ALDH2*1/*2 or ALDH2*2/*2 genotype was reported based on a comparison of a HCC cohort of 208 cases and a control cohort of 208 control cases conducted in Jiangsu, China 55. Moreover, a study of independent HCC cohorts suggested a negative correlation between ALDH2 expression and predisposition to malignant HCC 13. However, the correlation of the ALDH2*1/*1 genotype with increased HCC risk in HBV-positive patients with cirrhosis was suggested based on an analysis of 4155 HBsAg-seropositive participants, but no causal relationship was found between the ALDH2*1/*2 or ALDH2*2/*2 versus the ALDH2*1/*1 when alcohol drinking habits were considered 91.

The gene expression data from the Oncomine database showed that* ALDH1A1* expression was higher and *ALDH1B1* expression was lower in HCC tissues compared with normal tissues. Intrahepatic ALDH2 expression was lower at both the mRNA and protein levels in HCC and metastasis-inclined tissues compared with corresponding normal tissues and metastasis-averse tissues. A recent proteogenomic analysis of HBV-related HCC in China indicated an impaired liver-specific metabolic function in HCC cells and downregulation of ALDH1B1, ALDH2, and ALDH3B1 92. Proteomic clustering identified three subgroups, among which, the metabolism subgroup was characterized by the highest expression levels of proteins related to metabolism and liver function, including ALDH1A1, ALDH1A2, ALDH2, ALDH4A1, and ALDH9A1 92. In terms of prognosis, a lower ALDH2 level was a poor prognosticator following primary resection, coincident with older age, embolus, larger tumor size, extrahepatic metastasis and microvascular invasion in HCC patients 13. Furthermore, a correlation of higher *ALDH1B1* and *ALDH1L1* gene expression with better clinical outcomes was noted in HBV-related HCC patients 16, 93.

ALDH2 is implicated in alcohol-related cancers. As reported in a recent study in both patients and mice, deficiency in ALDH2 correlated well with a higher risk for advancement of alcohol related-fibrosis to HCC 39. Large amounts of oxidized mitochondrial DNAs are released via extracellular vesicles (EVs) by *Aldh2*-deficient hepatocytes. The EVs can be taken up by neighboring HCC cells, which in conjunction with acetaldehyde, activate multiple oncogenic pathways (including C-Jun N-terminal kinase, signal transducer and activator of transcription 3, BCL-2, and transcriptional co-activator with PDZ-binding motif), and promote HCC carcinogenesis after chronic exposure to both carbon tetrachloride and alcohol 39. These studies suggested that a reduction in the genesis of EVs containing the oxidized mtDNA represents a possible therapeutic strategy for mitigating the risk of ALD-associated HCC in ALDH2-deficient individuals 39. Furthermore, the number of sister-chromatid exchange (SCE) events is elevated 2.3-fold in *Aldh2^-/-^* mice, and a single exposure to alcohol causes a 4-fold increase in SCE events, indicating that resultant DNA damage activates recombination repair in response to the accumulation of endogenous aldehydes 94. It is known that genomic instability is a hallmark of cancer cells, as it generates a high frequency of DNA damage 95. Moreover, ALDH2 impacts metastasis largely through modulating the ALDH2-acetaldehyde -redox-AMP-activated protein kinase (AMPK) axis, because AMPK modulates lipid metabolism to regulate tumor cell growth and survival. Moreover, ectopic expression of ALDH2 ameliorates HCC metastasis both *in vitro* and *in vivo* 13, 96.

Furthermore, the overexpression of ALDHs (mainly ALDH1, ALDH3A1, and ALDH18A1) confers cancer cells with a survival advantage because oxidative stress resulting from high metabolic activity leads to ROS generation, lipid peroxidation, and the accumulation of toxic aldehydes, which can inhibit the proliferation and survival of cancer cells 97. As antioxidants, ALDHs can also decrease immunogenic cell death and limit tumor immunity by reducing endoplasmic reticulum stress and ROS production 98. As reported, *ALDH1B1* activity was downregulated in liver cancer tissues compared to normal liver tissue, and the reduced *ALDH1B1* activity was considered protective in HCC patients through its influence on the oxidation of short-chain aldehydes including acetaldehyde and propionaldehyde 16, and its protective against alcohol-induced hepatocellular proliferation and hepatic neoplasm in mice 99. In addition, ALDH1L1, which is also reduced in HCC, also can suppress cancer cell proliferation by depleting intracellular 10-formyltetrahydrofolatedehydrogenase, which is required for *de novo* purine biosynthesis 100, 101. ALDH3A1 was reported to be significantly upregulated in HCC and adenoma, resulting in the activation of the Wnt/β-catenin pathway and CTNNB1 mutations, and ALDH3 may slow the growth of HCC cells and inhibit the formation of aldehydes derived from lipid peroxidation 102, 103. In tumor immunity, the ALDH1 family (including ALDH1A1, ALDH1A2, and ALDH1A3) converts retinal to RA, which promotes the induction, function and stability of regulatory T cells, leading to immune tolerance that compromises tumor immunity 104.

### ALDH and Cancer Stem Cells

In liver carcinogenesis, some rarefied tumorigenic cells are considered as cancer stem cells (CSCs). These cells may have unlimited proliferative potential and may drive the formation, growth, and relapse of tumors. ALDHs are markers of cancer stem cells (CSCs) in a variety of cancers, and they modulate cell proliferation, metastasis, stem cell differentiation, and resistance to chemotherapeutic agents by protecting cells from toxins and differentiation-inducing stimuli 97, 105. CD133^+^/ALDH^high^ HCC cells, a subpopulation of cancer-initiating cells, were suggested to possess a high tumor-forming ability 106. Both CD133 and ALDH as markers can be used to identify dual-positive HCC cells, which may serve as a cell model to understand HCC carcinogenesis and to identify and select new therapeutic targets through molecular, genomic, and epigenetic analyses 106, 107. However, none of the commonly used markers (including CD133, ALDH, CD44, EpCAM, or CD90) can standalone as an ubiquitous marker for hepatic CSCs due to heterogeneity of the tumoral process 108. Additionally, ALDH was used as a biomarker for distinguishing normal from CSCs, making it a powerful predictor of poor clinical outcome 105, 109-111. However, a recent study demonstrated that deletion of the *Aldh1a* gene did not affect LPC proliferation or HCC progression 21. Therefore, further studies are required to clarify the role of ALDH in the control of LPCs.

**Figure 3:**
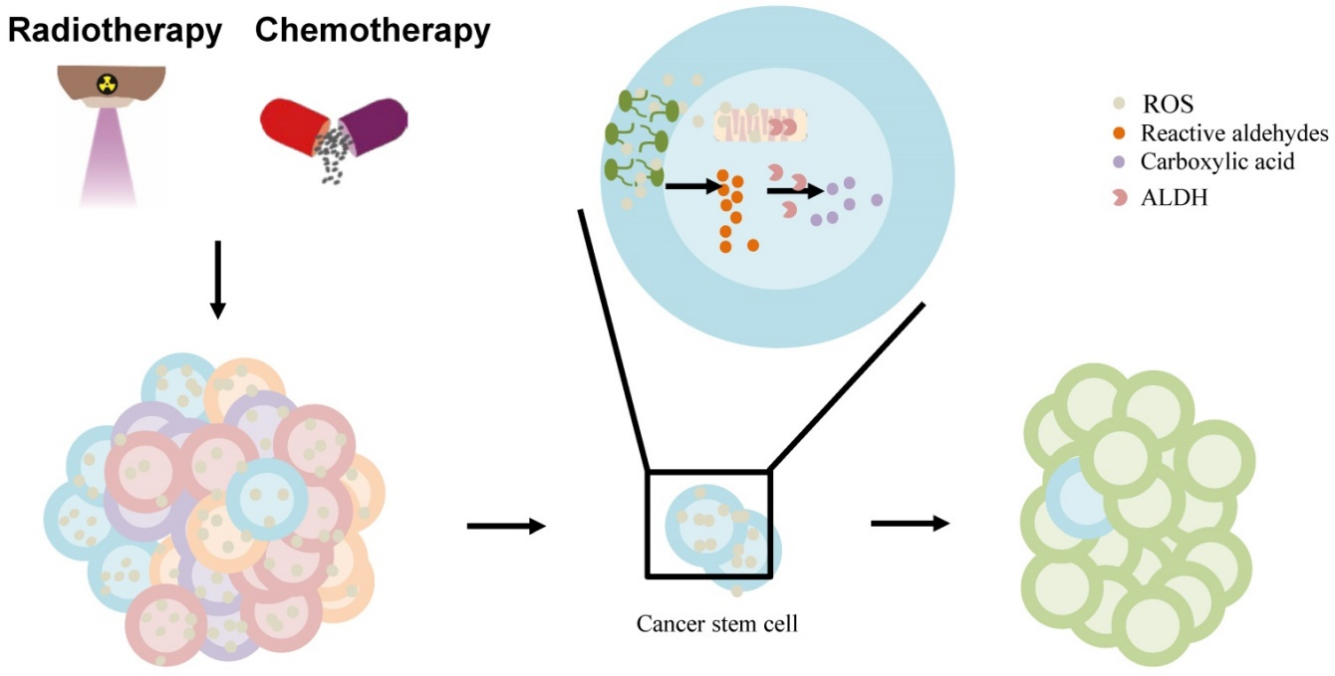
The effect of ALDH in liver cancer stem cell (CSC) model. The chemo-/radiotherapy leads to increased ROS, lipid peroxidation in cancer cells. ROS can provoke oxidation of PUFAs in membrane lipid bilayers to form highly reactive aldehydes. ALDH is involved in the oxidation of aldehydes into carboxylic acid in cancer stem cells, contributing to resistance and relapse. Abbreviations: ALDH, aldehyde dehydrogenase; CSC, cancer stem cell; PUFAs, polyunsaturated fatty acids; ROS, reactive oxygen species.

Furthermore, the standard chemotherapy and radiation treatments preferentially target non-CSCs, while CSCs are resistant to these treatments, which contributes to relapse after treatment. ALDHs, highly expressed in CSCs contribute to the chemotherapy- and radiotherapy-resistance 97. Owing to their increased metabolic activity and the use of radiation or ROS-generating drugs to treat them, cancer cells may experience significantly high oxidative damage, leading to increased ROS, lipid peroxidation, and the accumulation of toxic aldehydes, such as MDA, 4-hydroxy-hexenal, and 4-HNE 97, 112. ALDH activity may, under the regulation of intracellular scavenging, protect normal and cancer cells from injury by reducing ROS during chemo-/radiotherapy 113. Moreb *et al.* suggested that the resistance to chemo-/radiotherapy by CSCs can be mitigated by targeting ALDH proteins 114. ALDH, once hijacked in CSCs, oxidizes aldophosphamide to carboxyphosphamide and provides resistance to cyclophosphamide and other chemotherapeutic drugs (including temozolomide, 4-hydroperoxycyclophosphamide, irinotecan, paclitaxel, epirubicin, and doxorubicin) 111. Further, ALDH can reduce ROS and limit immunogenic cell death and antitumor immunity by interfering with the efficacy of ROS-generating drugs including anthracyclines, mitoxantrone, bleomycin, bortezomib, and cyclophosphamide 98 (Figure 3). Co-targeting of ALDH with other therapies might reduce resistance and improve the management of these cancers. ALDH inhibitors are classified into multi-ALDH isoform inhibitors and isoform-specific inhibitors, which can be utilized for cancer treatment (reviewed by Dinavahi *et al*.) 97. However, HCC cells lost ALDH2 protein expression compared to non-tumor tissues 13. Thus, further research is needed to determine whether ALDH inhibitors can be used to treat liver cancer patients in combination with other cancer therapies.

## Therapeutic Prospects

ALDH2 is the key enzyme that metabolizes acetaldehyde and is a great therapeutic target for the treatment of alcoholism. An estimated 8% of the world's population, consisting mainly of individuals of East Asian descent, have the ALDH2*2 allele, which encodes a nonfunctional ALDH2 protein. In general, those with the ALDH2*2 allele consume less alcohol, but some still drink a large amount of alcohol, causing accumulation of acetaldehyde in the body. The roles of acetaldehyde in liver inflammation, fibrosis, and cancer development still remain obscure. Further studies are required to determine whether ALDH2 can serve as a therapeutic target for the prevention and treatment of liver disease and cancer. Moreover, considering the significant role of ALDH2 in drug metabolism, an altered ALDH2 function may also lead to significant changes in the pharmacokinetics of substrate drugs and therefore introduce potential variable drug efficacy or adverse events. Therefore, we need to learn more about the effects and particularity of ALDH2 polymorphisms, especially in alcoholic subjects, to offer them appropriate dosing adjustments and help them make well-informed treatment choices in the future.

## Figures and Tables

**Table 1:** Human ALDH families and their functions.

ALDH Family	Chromosomes	Functions	References
ALDH1A1	9q21.13	Metabolizes retinal to RA; oxidizes acetaldehyde, LPO-derived aldehydes, DOPAL; protects against ultraviolet-induced damage as lens and Corneal crystallins; mediates a GABA synthesis pathway	14, 115-117
ALDH1A2	15q21.3	Metabolizes retinal to RA; oxidizes acetaldehyde and LPO-derived aldehydes	118
ALDH1A3/ALDH6	15q26.3	Metabolizes retinal to RA; oxidizes LPO-derived aldehydes	118
ALDH1B1/ALDH5	9p13.1	Oxidizes acetaldehyde and LPO-derived aldehydes	118, 119
ALDH1L1/FDH	3q21.3	Converts 10-fTHF to tetrahydrofolate	120
ALDH1L2/mtFDH	12q23.3	Converts 10-fTHF to tetrahydrofolate	121
ALDH2	12q24.12	Metabolizes acetaldehyde, DOPAL, and LPO-derived aldehydes; acts as a nitrate reductase	118
ALDH3A1	17p11.2	Oxidizes aromatic, aliphatic aldehydes, and LPO-derived aldehydes; protects the cornea and lens against ultraviolet-induced oxidative stress	117
ALDH3A2/FALDH	17p11.2	Oxidizes fatty aldehydes	118
ALDH3B1	11q13.2	Oxidizes LPO-derived aldehydes; involves in an alteration of dopamine metabolism	118
ALDH3B2	11q13.2	Unknown	
ALDH4A1/P5CD	1p36.13	Oxidizes glutamate γ-semialdehyde; Oxidizes short- and medium-chain aliphatic LPO-derived aldehydes	115
ALDH5A1/SSADH	6p22.3	Metabolizes succinic semialdehyde to succinate	115
ALDH6A1/ MMSDH	14q24.3	Oxidizes malonate, methylmalonate semialdehyde and malondialdehyde; invloves in valine and pyrimidine catabolism	115, 118
ALDH7A1/EPD	5q23.2	Oxidizes LPO-derived aldehydes, alpha-aminoadipic semialdehyde, and betaine aldehyde	122
ALDH8A1	6q23.3	Metabolizes retinal to RA; oxidizes acetaldehyde and LPO-derived aldehydes	118
ALDH9A1	1q24.1	Metabolizes γ-Aminobutyraldehyde, betaine aldehyde and catecholamine-derived aldehydes	118
ALDH16A1	19q13.33	Probably takes part in the etiology of gout	123
ALDH18A1/P5CS	10q24.1	Catalyzes the reduction of glutamate to Δ-pyrroline-5-carboxylate	118

Abbreviations: DOPAL, 3,4-dihydroxyphenylacetaldehyde; FALDH, fatty aldehyde dehydrogenase; FDH, 10-formyltetrahydrofolate dehydrogenase; GABA, γ-aminobutyric acid; LPO, lipid peroxidation; MMSDH, methylmalonate semialdehyde dehydrogenase; P5CD, pyrroline-5-carboxylate dehydrogenase; P5CS, Δ-pyrroline-5-carboxylate synthase; RA, retinoic acid; SSADH, succinic semialdehyde dehydrogenase; 10-fTHF, 10-formyltetrahydrofolate.

## Abbreviations

ALDH2: acetaldehyde dehydrogenase 2
ALD: alcoholic liver disease
AMPK: AMP-activated protein kinase
AGS: acetaldehyde-generating system
ALC: alcoholic liver cirrhosis
CYP2E1: cytochrome p450 2E1
CSCs: cancer stem cells
ECM: extracellular matrix
EVs: extracellular vesicles
HCC: hepatocellular carcinoma
HBV: hepatitis B virus
HCV: hepatitis C virus
4-HNE: 4-hydroxy-nonena
HSPCs: hematopoietic stem and progenitor cells
HSC: hepatic stellate cell
ISGs: interferon-stimulated genes
LPCs: liver progenitor cells
MAA: malondialdehyde-acetaldehyde
MitoQ: mitochondria-targeted lipophilic ubiquinone
MMP: matrix metalloproteinase
MitoQ: mitochondria-targeted lipophilic ubiquinone
NAFLD: non-alcoholic fatty liver disease
NAD: nicotinamide adenine dinucleotide
NASH: non-alcoholic steatohepatitis
PP2A: protein phosphatase 2A
PPARγ: peroxisome proliferator-activated receptor gamma
PIAS-1: protein inhibitor of activated STAT-1
ROS: radical oxidative species
RA: retinoic acid
ROL: retinol
SNPs: single nucleotide polymorphisms
SCE: sister-chromatid exchange
STAT: signal transducer and activator of transcription
SREBP1c: sterol regulatory element-binding protein 1c
TGF-β1: transforming growth factor beta 1
